# A Review of metabolic staging in severely injured patients

**DOI:** 10.1186/1757-7241-18-27

**Published:** 2010-05-17

**Authors:** Maria-Angeles Aller, Jose-Ignacio Arias, Alfredo Alonso-Poza, Jaime Arias

**Affiliations:** 1 Surgery I Department, School of Medicine, Complutense University of Madrid, Madrid, Spain; 2 General and Digestive Surgery Unit, Monte Naranco Hospital, Consejeria de Salud y Servicios Sanitarios, Principado de Asturias, Oviedo, Spain; 3 General and Digestive Surgery Unit, Sudeste Hospital, Arganda del Rey, Madrid, Spain

## Abstract

An interpretation of the metabolic response to injury in patients with severe accidental or surgical trauma is made. In the last century, various authors attributed a meaning to the post-traumatic inflammatory response by using teleological arguments. Their interpretations of this response, not only facilitates integrating the knowledge, but also the flow from the bench to the bedside, which is the main objective of modern translational research. The goal of the current review is to correlate the metabolic changes with the three phenotypes -ischemia-reperfusion, leukocytic and angiogenic- that the patients express during the evolution of the systemic inflammatory response. The sequence in the expression of multiple metabolic systems that becomes progressively more elaborate and complex in severe injured patients urges for more detailed knowledge in order to establish the most adequate metabolic support according to the evolutive phase. Thus, clinicians must employ different treatment strategies based on the different metabolic phases when caring for this challenging patient population. Perhaps, the best therapeutic option would be to favor early hypometabolism during the ischemia-reperfusion phase, to boost the antienzymatic metabolism and to reduce hypermetabolism during the leukocytic phase through the early administration of enteral nutrition and the modulation of the acute phase response. Lastly, the early epithelial regeneration of the injured organs and tissues by means of an oxidative metabolism would reduce the fibrotic sequelae in these severely injured patients.

## Introduction

Inflammation is a complex, multiscale biologic response to stress that is also required for repair and regeneration after injury [1]. Particularly, in patients with severe accidental or surgical trauma the inflammatory response shows its multifaceted and actually soundless capacity [2-5].

In the last century, David P. Cuthbertson [6], Hans Selye [7] and Francis D. Moore [8] attributed a meaning to the post-traumatic inflammatory response accordingly with previous discoveries and the knowledge of the time. By using teleological arguments, these extraordinary authors tried to make inroads into the understanding of the metabolic response of the body to injury [6-8]. Their interpretations of this response, not only facilitates integrating the knowledge, but also the flow from the bench to the bedside, which is the main objective of modern translational research [1,9,10].

Thus, this would justify the contribution of new interpretations of the metabolic response to injury, in an attempt to facilitate incorporating the newly acquired knowledge of these conditions, in addition to other apparently disparate diseases that have common biological pathways and therapeutic approaches [9].

### Trophic mechanisms linked to the evolution of the Inflammatory Response

We have formulated the hypothesis that both acute local and systemic inflammatory response to injury are based on the successive pathologic functional predominance of three systems referred to as the nervous, inmune and endocrine systems. These names are based on the idea that the final and prevalent functions traditionally attributed to these systems may represent the consecutive response phases to stress [11-13].

This hypothesis implies that the successive pathophysiological mechanisms developed by the body when undergoing inflammation are considered increasingly complex trophic functional systems for using oxygen [12,13].

**Figure 1:**
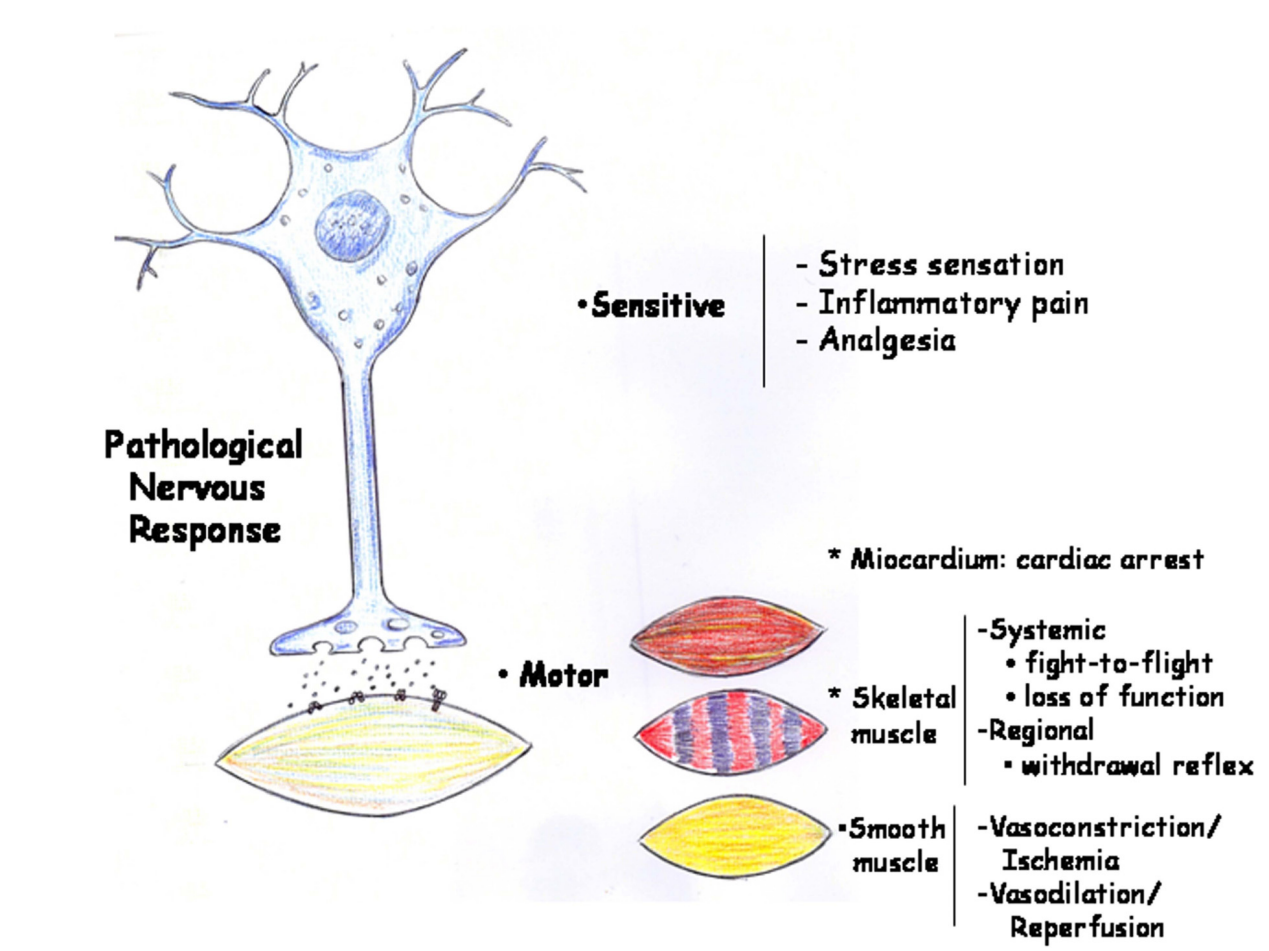
**Ischemia/Reperfusion phenotype**. Schematic representation of the Pathological Nervous Response in the severe traumatized patient.

The first or immediate phase hase been referred to as the nervous phase, because the sensory (pain and analgesia) and motor (contraction and relaxation) alterations respond to the injury. The nervous or immediate functional system presents ischemia-revascularization and edema, which favor nutrition by diffusion through the tissues and organs. This trophic mechanism has a low energy requirement that does not require oxygen (ischemia) or in which the oxygen is not correctly used, with the subsequent development of oxidative and nitrosative stress. In this phase, while the progression of interstitial edema increases the space between the parenchymal cells and the capillaries, the lymphatic circulation is simultaneously activated (circulatory switch). Thus, tissues and organs adopt an ischemia-revascularization phenotype [12,13] (Figure 1).

In the following immune or intermediate phase of the inflammatory response, the tissues and organs which have suffered ischemia-reperfusion are infiltrated by inflammatory cells and bacteria. Symbiosis of these cells and bacteria for extracellular digestion by enzyme release (fermentation) and by intracellular digestion (phagocytosis) could be associated with a hypothetical trophic capacity. Improper use of oxygen persists in this immune phase and is also associated with enzymatic stress. Furthermore, lymphatic circulation plays a major role while macrophages and dendritic cells migrate to lymph nodes where they activate lymphocytes. As a result, tissues and organs adopt an leukocytic phenotype [12,13].

Angiogenesis characterizes the last or endocrine phase of the inflammatory response, during which nutrition mediated by the blood capillaries is established. However, the angiogenic process becomes active early and excessive proliferation of endothelial cells takes place which, in turn, develops a great density of endothelial sprouts [14]. Though this initial and excessive proliferation, the endothelial cells could successively perform antioxidant and antienzymatic functions. These functions would favor the evolution of the inflammatory response towards tissue repair through specialized capillary development. Then, it would be in this last phase of the inflammatory response when the process of angiogenesis would be responsible for tissue nutrition through capillaries. Oxygen and oxidative metabolism are an excellent combination through which cells can obtain abundant energy (energetic stress) for tissue repair by regeneration or wound healing. As a result, tissues and organs adopt an angiogenic phenotype [13,14].

The sequence in the expression of progressively more elaborated and complex nutritional systems could hypothetically be considered the essence of the inflammation regardless of its etiology (traumatic, hypovolemic or infectious) or localization. Therefore, the incidence of harmful influences during their evolution could involve regression to the most primitive trophic stages, in which nutrition by diffusion (ischemia-reperfusion phenotype) takes place. Moreover, the incidence of noxious factors during the evolution of the systemic inflammatory response produces severe hemodynamic alterations again, and lastly vasodilatory shock, with tissue hypoxia, hypothermia and acidosis, is established. This mechanism of metabolic regression is simple, and also less costly. It facilitates temporary survival until a more favorable environment makes it possible to initiate more complex nutritional ways to survive (leukocytic and angiogenic phenotypes) [12,13].

### Phases of the metabolic response to the injury

Severe injury induces a systemic inflammatory response in the body that could be developed through the expression of three successive phenotypes: Ischemia-reperfusion phenotype, leucocytic phenotype and angiogenic phenotype. In turn, it has already been proposed that these phenotypes could represent the expression of trophic functional systems of increasing metabolic complexity [12,13]. This hypothetical approach to the mechanisms that govern the systemic inflammatory response could be based on the increasing metabolic ability of the body to use oxygen over the successive phases of its evolution towards the tissue repair. Therefore, in the severe injury trauma, it could be considered that the body adapts the support (trophic system) to the metabolic needs characteristic of each inflammatory phenotype, regardless of the energy type involved in its production (mechanical, thermic, electric or nuclear). In turn, the metabolic ability of each phenotype would be determined by the mechanisms used for cellular energy production.

### Metabolism related to Ischemia-Reperfusion phenotype

This phenotype would characterize the immediate or nervous phase of the systemic response to the injury. The nervous alterations in this phase, both sensitive (sensation of stress, inflammatory pain and analgesic response) and motor (contraction and relaxation) predominate. The latter alterations would correspond to both the myocardium (arrhythmias, cardiac arrest), and either the skeletal or voluntary muscle (fight-or-flight response, withdrawal reflexes, loss of motor function) or the smooth involuntary muscle (vasoconstriction with ischemia-reperfusion) related to vasodilation, shock and reperfusion injury [13].

**Figure 2:**
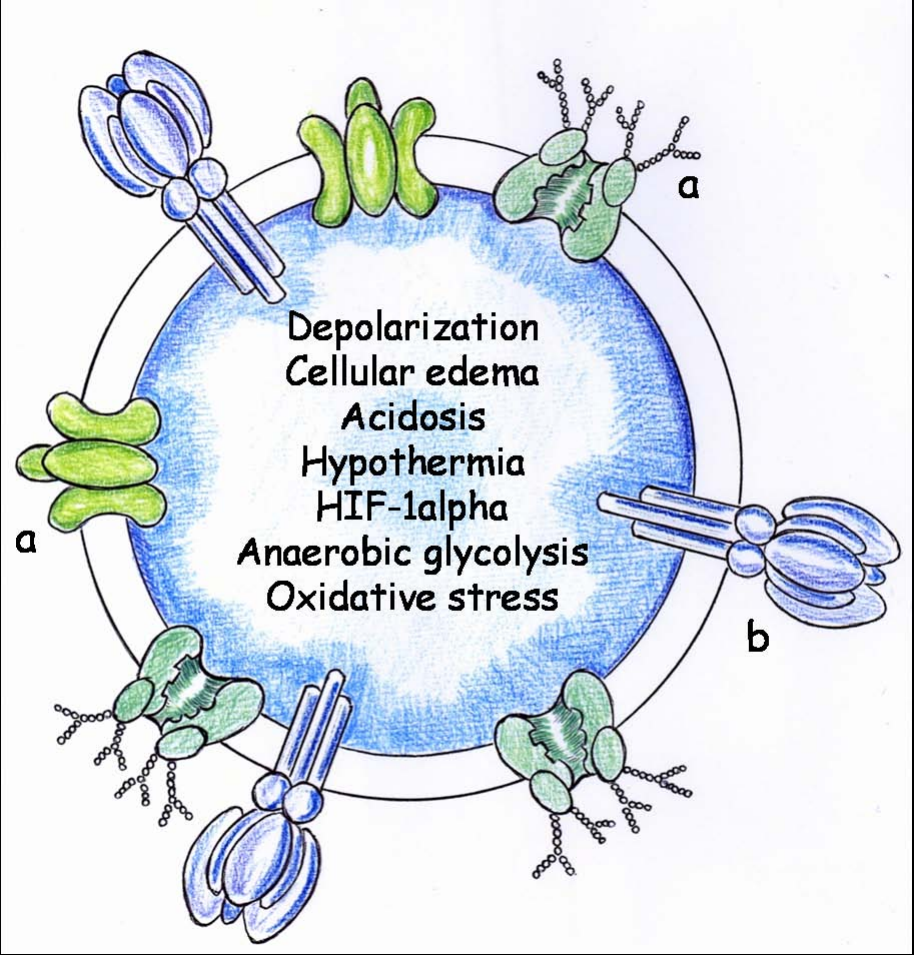
**Cellular phenotype of Ischemia/Reperfusion**. When this phenotype is expressed it produces a body hydroelectrolitic redistribution. Thus, cellular and interstitial edema is developed. a: Sodium-Potassium pump. b: Calcium-ATPase pump.

A common pathogenic mechanism of this neuromuscular response would be the sudden alteration of cellular membrane potential with depolarization. Thus, there is increasing evidence that conditions characterized by an intense local or systemic inflammatory response are associated with abnormal ion transport [15]. Early and pathological changes in ion transport in neuromuscular cells could therefore initiate the inflammatory response. In addition, disturbances of ion transport have been associated with intra and extracellular edema [13,15] (Figure 2).

It has been proposed that both cellular as well as interstitial edema could represent an ancestral mechanism to feed cells by diffusion [12,13]. Consequently small fluctuations in cell hydration can act as separate and potent signals for cellular metabolism and gene expression. Most importantly, cell volume changes can be secondary to cumulative substrates and hormones uptake [16]. Based on this idea, activation of the hypothalamic-pituitary-adrenal axis and the adrenomedullary system with glucocorticoid secretion and release of epinephrine into the circulation, which occurs in the early evolutionary period [17], causes the selective accumulation of these substances in the interstitial space of the tissues and organs that suffer ischemia-reperfusion because their endothelial permeability is increased [13].

In this initial phase of the inflammatory response, it could be considered that hypometabolism, anaerobic glycolisis with lactate production, low temperature and a decrease in energy expenditure could be related to a primitive cellular trophic mechanism that may be favored by neuro-endocrine stress-response substances. Interestingly enough, the functional impotence of the somatic motor system, which controls voluntary movements, favors blood stasis and interstitial edema [13].

The edema associated with a depression of the metabolism has been termed by David Cuthbertson, as the "ebb phase" [6,18]. This phase is characterized by hemodynamic instability [19] and is described in classical literature on trauma as the period of shock [6].

The direct mitochondrial inhibition by nitrogen and oxygen species coupled with reduced hormonal stimulation and decreased positive feedback from decreased metabolic demands all combine to reduce energy production. It is supposed that through this mechanism the affected cells enter in a dormant stage analogous to hibernation or aestivation [20]. That is why this metabolic pathway is considered a potentially protective mechanism because the reduced cellular metabolism could increase the chance of survival of cells, and thus organs, in the face of an overwhelming injury [20].

Hibernation is considered to be a retained ancestral trait in modern mammals. Whether hibernation is inherited or a newly developed trait, the widespread distribution of mammalian species that hibernate suggest that the genes required to specify the hibernating phenotype are common among the genomes of all mammals [21]. Therefore "cell hibernation" or "cell stunning" during ischemia could be a situation of dedifferentiation, with an intention to adapt to the imposed changes. In this situation specialized functions would not be expressed, although primitive metabolic pathways and the corresponding primitive trophic functions would be maintained [13].

In essence, the objective of the alterations produced during the expression of the ischemia-reperfusion phenotype could be to induce the highest metabolic autonomy of tissues and organs. Thus, the swollen interstitial space would become the storage area for those substances that are suddenly released into the blood circulation during the response to stress [7]. In this way, raised blood concentrations of catecholamines and stress hormones, such as cortisol [7,17,22] and glucagon [23], growth hormone and prolactine [24], glucose and glycolytic intermediates i.e. lactate and piruvate [6], triglycerides, free fatty radicals and glycerol [6,24], amino acids as alanin, nitrogen [8,25] and sulphur [6,25] have been described. These substances stored in the interstitial space would facilitate the survival of the hypofunctional cells, allowing for their metabolic autonomy, so a normal state can be restored.

Anoxic environments have occurred throughout the Earth's history. The anoxic atmosphere of the primitive earth probably contained water vapor, N_2_, H_2_, hydrogen sulfide (H_2_S), CO, CO_2_, HCN and CH_4_. However, as the level of O_2 _increased, so did the toxic effects of its one-electron reduction products, its highly reactive singlet reached proportions that made the development of efficient scavenging and protection systems necessary[26]. Sulfhydryl compounds (H_2_S, CH_3_, SH, cysteine, glutathione), antioxidants (carotenoids, vitamins C, A and E), an array of enzymes (catalase, superoxide dismutase, peroxidases) and thiol-rich proteins (thioredoxin, glutaredoxin) all became necessary as defenses against damage by reactive oxygen and nitrogen species [26]. H_2_S must have been the predominant antioxidant early in prokaryotic evolution [26,27].

Perhaps, by knowing these precedents it is not surprising that H_2_S may play a beneficial role in conditions associated with the increased generation of reactive oxygen and nitrogen species [28]. In particular H_2_S may be useful to prevent damage associated with hypoxia. Therefore mice exposed to H_2_S enter into a physiological state similar to that observed when animals initiate hibernation, daily torpor or aestivation, that allows them to endure periods of low metabolic rate and decreased core body temperature without apparent ill effects [29,30].

After a severe injury the cardiovascular response can move from cardiac arrest to shock. Since cardiac arrest is an evolutive injury, it has been suggested that the optimal treatment is phase-specific and includes: The electrical phase (0-4 minutes), the circulatory phase (4-10 minutes) and the metabolic phase (beyond 10 minutes after cardiac arrest) [31]. In any case, early initiation of cardiopulmonary resuscitation is the most effective measure [32].

However, other metabolic, i.e. hypothermia, and biochemical interventions, are likely to be effective in the metabolic phase of cardiac arrest. Two complementary ways to cover the management of the metabolic phase of cardiac arrest are considered. The first phase consists in reducing the adverse effects of metabolic cardiac arrest promoting basic research on prolonged global whole-body ischemia. The second phase aims at diminishing the tissue injury from reperfusion related to cardiopulmonary resuscitation, studying the optimal metabolic conditions of reperfusion, i.e. restoring oxygen and substrates [31].

Shock, regardless of etiology, is characterized by decreased delivery of oxygen and nutrients to the tissues. Our therapeutic interventions are directed toward reversing the cellular ischemia and preventing its consequences [33,34]. Reperfusion injury starts with the simple reoxygenation of tissues after ischemic insult [35]. Damage control resuscitation [33-36] and damage control surgery [36-39] are used in this early evolutive phase, in accordance with the patient's physiologic tolerance [39]. The principles of damage control have led to improved survival and have stopped bleeding until the physiologic derangement has been restored and the patient could undergo a prolonged operation for definitive repair [5,36-38]. Damage control avoids the "lethal triad" of hypothermia, acidosis and coagulopathy resulting in a vicious cycle that often cannot be interrupted [39,40].

It is currently accepted that the pathophysiological processes in the first days after injury seem to be important for the development and final outcome in patients with early multiple organ failure [41]. That is why reducing initial damage caused by the ischemia and/or the reperfusion would determine a more favorable evolution. Therefore, the two-hit hypothesis for the development of multi-organ dysfunction syndrome has been described to be caused either by the first hit, including organ and soft tissue injuries, as well as hypoxia and ischemia, or later due to a second or multiple hits, such as ischemia-reperfusion or surgical procedures [4,38,42].

Oxygen deprivation is an important determinant of cellular function during the expression of the ischemia-reperfusion phenotype [43]. The transcriptional response to hypoxia relies on multi-protein complexes to regulate several transcription factors, the best studied being hypoxia inducible factor (HIF). HIF is a heterodimer which enhances the expression of hypoxia responsive genes and therefore allows improved cell survival in situations of limited oxygen availability [43-45]. As a result, injured cells could turn to glycolisis to meet their energetic demands in hypoxia. Although glycolisis is less efficient than oxidative phosphorylation for producing ATP, the presence of sufficient glucose can sustain ATP production due to increased activity of glycolytic enzymes [46].

Post-traumatic hyperglycemia induced by catecholamines, among other factors, [6,19] would also favor the selective support of glucose and therefore the "glycolytic switch" in order to obtain ATP. In turn, glycolytic metabolism end products, i.e. pyruvate and oxaloacetate, can promote HIF-1α protein stability and activate HIF-1 inducible gene expression [45]. In addition to impairing cellular energy metabolism, hypoxia leads to differentiation inhibition and maintains the undifferentiated cell state [47].

Furthermore, the excess production of reactive oxygen and nitrogen species in this phase would cause oxidative stress, which would in turn result in bond cleavage and lipid and protein molecular breakdown, whose final products would become substrates in cases of extreme need [12,13]. Lastly, oxidative stress is one of the principal factors inducing the expression of the nuclear factor Kappa B (NF-κB) [44].

Tissue reoxygenation is mediated by oxygenase. In particular, carbon monoxide (CO) is one of the three products of heme degradation by heme oxygenase (HO)-1. Essentially nothing is known about local concentrations of CO that are achieved in vivo and whether that CO produced endogenously has a therapeutic effect [48]. Another gas, nitric oxide (NO), has been involved as a tissue protective agent during ischemia-reperfusion. NO seems to protect cells by attenuating the oxidant stress that occurs during ischemia by inhibiting an oxidase system initiated during ischemia which becomes amplified during the reperfusion phase. In addition, NO can lessen oxidative injury by scavenging reactive oxygen molecules [49].

Resuscitation is related to microcirculatory distress. Microcirculatory failure can occur in the presence of normal or supranormal systemic hemodynamic- and oxygen-derived variables, with microcirculatory distress being masked from the systemic circulation by shunting pathways [50]. In particular, splanchnic microcirculatory dysfunction can produce gastrointestinal tract hypoxia or dysoxia, a state in which the O_2 _supply is inadequate to meet tissue metabolic needs [51]. So, by the great vulnerability of splanchnic blood flow [52], the first hit, usually ischemia, results in a gastrointestinal tract priming, rendering it more susceptible to a secondary challenge i.e. reoxygenation, that stimulates an inappropriate inflammatory response [53]. Therefore, the changes in the intestinal microcirculation are in concert with the "two-hit" theory for multiple organ failure [53,54], which would at the same time confirm the proposal by Metchnikoff that the engine behind multiple-organ-failure syndrome is the gastrointestinal tract [55].

### Metabolism related to leukocytic phenotype

This phenotype would characterize the intermediate or immune phase of the systemic response to the injury. In this phase the tissues and epithelial organs, which have previously suffered ischemia-reperfusion, are infiltrated by inflammatory cells and bacteria. This infiltration occurs in an edematous oxygen-poor environment [13].

In these tissues and organs, which show oxidative and nitrosative stress, symbiosis of the inflammatory cells and bacteria for extra- and intra-cellular digestion could be associated with a hypothetical trophic capacity [12,13] which is why their metabolic autonomy would persist in this phase.

The metabolic response to injury in this immune phase of the inflammatory response is characterized by hypercatabolism and hypermetabolism [8,19,24,56]. This phase corresponds to the post-shock catabolic response or hypermetabolic flow phase of Cuthbertson [6,18,23].

The hypermetabolic response after a severe injury has been described as a hyperdynamic response with increased body temperature, oxygen and glucose consumption, CO_2 _production, glycogenolysis, lipolysis, proteolysis and futile substrate cycling [18,23,24,56-58]. The consequences of hypermetabolism are a great loss of body weight associated with a tremendous loss in essential body structures [58].

However, hypermetabolism is further associated with immunologic [2,4,38,59] and endocrinologic responses [2,59,60]. The immuno-inflammatory response is initiated immediately following injury and is mainly regulated by cytokines, which act as communication mediators between leukocytes, bridging the innate and adaptive immune response [38]. The immune response leads to systemic inflammatory response syndrome or SIRS, followed by a period of recovery mediated by counter-regulatory anti-inflammatory response (CARS) [2,4,38,59,61].

**Figure 3:**
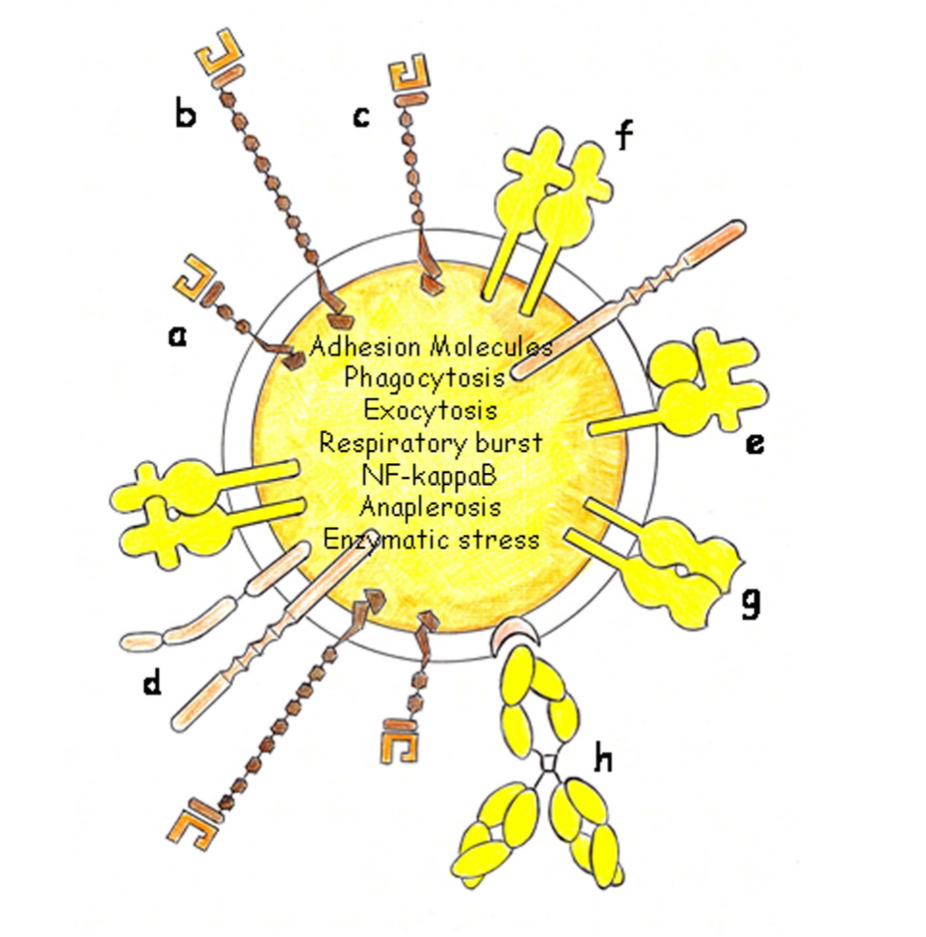
**Cellular Leukocytic Phenotype**. Adhesion molecules are overexpressed on the cells surface favoring the leukocytes and bacteria translocation. a: L-Selectin; b: P-Selectin; c: E-Selectin; d: Integrin; e: HLA-Class I molecule; f: HLA-Class II molecule; g: HLA-Class II receptor; h: Immunoglobulin.

Activated phagocytes, i.e. granulocytes and monocytes, would require anaerobic glycolysis as the main source of ATP for their functions [62]. This suggests that activated leukocytes are able to metabolically adapt to the hypoxic environment in this evolutionary phase of inflammation. Thus, it was shown that HIF-1α is essential for the upregulation of enzymes of the glycolytic pathway to supply phagocytes with enough levels of ATP [62]. Also the oxidative burst is part of the physiological function of phagocytes connected to massive production and release of reactive oxygen species and respiratory burst [63]. Inability of the mitochondria to use oxygen due to the uncoupling of the electron chain transport is a focus of ongoing research [35] (Figure 3).

Depression of leukocyte mitochondrial respiration secondary to the decrease in oxygen metabolism could induce obtaining energy by other mechanisms. For example, phagocytes could generate sufficiently reduced nicotinamide dinucleotide phosphate (NADPH) for their biological functions, through the continuous replenishment of Krebs cycle intermediates [64]. Through these anaplerotic mechanisms, phagocytes could obtain sufficient energy not only for the new functions acquired but also for proliferation. These mechanisms, similar to those used by cancer cells, would also allow leukocytes to maintain a metabolic phenotype of biosynthesis aside from the normal physiological constrains and therefore, would acquire an increased metabolic autonomy [65].

Based on this metabolic similarity to cancer cells, glutamine, the most abundant amino acid in mammals, could be used to replenish the tricarboxylic acid cycle of leukocytes during this immune phase of the systemic inflammatory response [64]. It would then be explained that the administration of this non-essential amino acid induces an immunoestimulatory effect [38] in these severe injured patients with a marked acute and prolonged depletion of intracellular glutamine [66]. Thus, NF-κB is known to be a redox sensitive transcription factor with regard to the production of pro-inflammatory molecules including chemokines, cytokines and adhesion molecules, which allow leukocytes to attach themselves to the endothelium and facilitate their extravasation to the interstitial space of tissues and organs [35,67].

The metabolic autonomy of leukocytes would also be reflected in its ability for pro-opiomelanocortin (POMC) production [64]. POMC is processed in the anterior lobe of the pituitary gland into an N-terminal fragment, corticotropin (ACTH) and β-lipotrophic hormone (LPH), while the intermediate lobe produces γ-melanocyte-stimulating hormone (MSH), α-MSH, corticotropin-like intermediate lobe peptide (CLIP), γ-lipotropin (LPH) and β-endorphin. An interesting aspect of leukocyte POMC production is that, while the same peptides are produced as in the pituitary, the pattern varies, and some are unique to leukocytes [68].

Corticotropin releasing factor (CRF), which is released from the hypothalamus during stress, is also produced by leukocytes and within its action, pro-inflammation, through the enhancement of the NF-κB intracellular signaling pathway, stands out [69].

**Figure 4:**
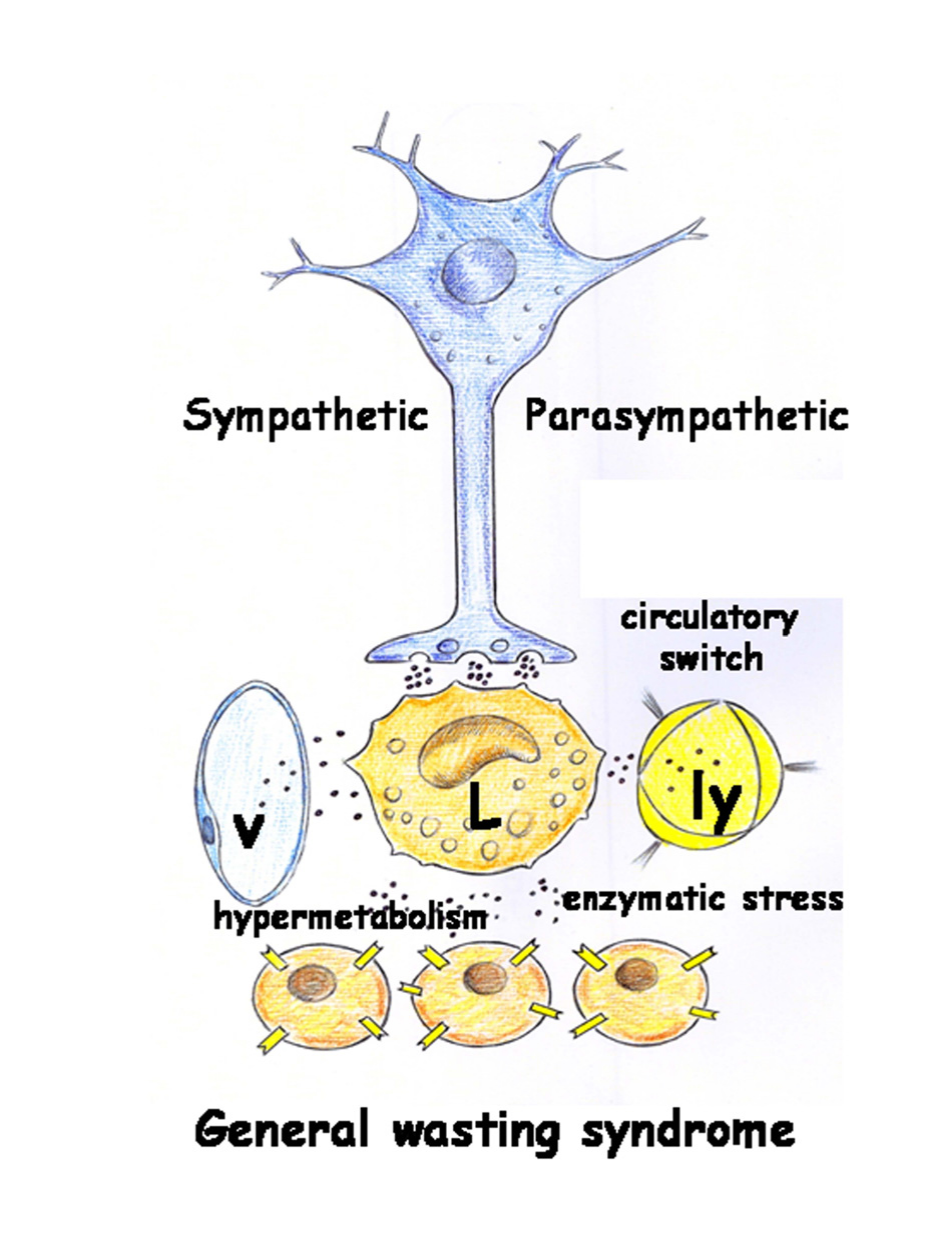
**Schematic representation of the neuro-immune-endocrine capacity of the leukocytic (L) phenotype**. The immune cells are considered a new diffusely expressed adrenergic organ as well a peripheral neuroendocrine system with autocrine and paracrine functions. In addition, inflammatory mediators released by leukocytes induce a circulatory switch which favors the lymphovenous circulation. Lastly, the leukocytic phenotype is associated with enzymatic stress and hypermetabolism which, in turn, cause a General Wasting Syndrome. v: venous vascular system; ly: lymphatic vascular system; p: parenchymal cells.

Immune cells are also considered a new, diffusely expressed adrenergic organ, and they have the ability to generate release and degradate catecholamines. It seems that catecholamines use intracellular oxidative mechanisms to exert autoregulatory functions on immune cells [70]. The physiological counterpart of the adrenergic system, the cholinergic system, is also known to be an integral part of human macrophage and lymphocyte regulation and is termed the "cholinergic anti-inflammatory pathway". In this pathway, the efferent activity in the vagus nerve releases acetylcholine (ACh), which interacts specifically with macrophage α_7 _subunits of nicotinic ACh receptor, leading to cellular deactivation and inhibition of pro-inflammatory cytokine release [71]. Therefore, leukocytes through their neurohormonal capacity could modulate the immunological response inducing even hyperinflammation or inmmunoparalysis [2,61,72,73]. In essence, leukocytes seem to become a peripheral neuroendocrine system, with autocrine and paracrine functions. This would constitute an alternative to the central neuroendocrine system, with a predominantly endocrine function, represented by the hypothalamic-pituitary-organ hormonal axes. These are suppressed in prolonged critical illness and contribute to the general wasting syndrome [74,75] (Figure 4).

The immune response underlying the expression of the leukocyte phenotype could also have a gastrointestinal origin. The gastrointestinal tract mucosa contains the largest reservoir of macrophages in the body. As effecter cells, intestinal macrophages, together with mast cells, are first-line defense mechanisms [76]. If this defense capacity is overtaken, the intestine in the critically ill surgical patient becomes an "undrained abscess" [77] and the pathological gastrointestinal colonization is associated with the development of infection and sepsis with late multi-organ failure [78]. Also, during the expression of this immune response, the lymphatic splanchnic circulation would acquire increasing importance [79,80]. Therefore, the expression of the leukocyte phenotype in the intestine could possibly change this organ into the most important peripheral autonomous neuroendocrine system, in view of the large accumulation of leukocytes with metabolic autonomy and neuroendocrine capacity.

### Metabolism related to angiogenic phenotype

This phenotype would characterize the late or endocrine phase of systemic response to injury. In this phase, a return to the prominence of oxidative metabolism would be produced, and therefore angiogenesis, in the affected tissues and organs to create the capillary bed that would make regeneration possible or to carry out repair through fibrosis or scarring [12,13].

The endocrine functional system facilitates the arrival of oxygen transported by red blood cells and capillaries. It is considered that angiogenesis characterizes this last phase of the inflammatory response, in which nutrition mediated by the blood capillaries is established. The ability to use oxygen in the oxidative metabolism is restored when patients recover their capillary function and, as a result, nutrition mediated by the capillaries is also restored (endocrine or late phase).

This type of metabolism is characterized by a large production of ATP (coupled reaction) which is used to drive multiple specialized cellular processes with limited heat generation that would induce the onset of healing. In the convalescent phase, the previous dedifferentiated epithelia specializes again, the energy stores that supplied the substrate necessary for this demanding type of metabolism are replenished, and complete performance is reached, thus making active normal life possible [13,14].

Angiogenesis is defined as the growth of new vessels from preexisting ones [81]. Although the final objective of endothelial growth is to form new vessels for oxygen, substrates and blood cell transport (vascular phase), other functions could also be carried out before the new vessels are formed (prevascular phase). In the initial phases of the inflammatory response, the new endothelial cells formed could have a function associated with anti-inflammatory effects. That is, with anti-oxidative and anti-enzymatic stress properties, favoring the progression of the inflammation as well as its resolution [14].

Angiogenesis is critically dependent on vascular endothelial growth factor (VEGF) action. HIF-1α upregulates a number of factors involved in cytoprotection, including angiogenic growth factors such as VEGF, endothelial progenitor cell recruitment via the endothelial expression of stromal-derived factor SDF-1, HO-1 and erithropoietin [82]. Furthermore, VEGF promotes monocyte chemotaxis and the expression of adhesion molecules [43]. Also, when this last phase of inflammation begins, macrophages and lymphocytes become the predominant cell types within the injured tissue. Macrophages, in particular, adopt a potentially angiogenic phenotype [83-85]. Moreover, peripheral blood mononuclear cells can be differentiated into monocytes, lymphocytes and endothelial cells. Therefore, endothelial progenitor cells in the circulation may promote neoangiognesis and produce the spontaneous regeneration of the endothelium in the injured tissue [86].

In contrast to their role in promoting inflammation, the ability of alarmins to promote tissue repair and regeneration is of increasing interest. Importantly high mobility group box-1 (HMGB-1) induces migration of stem cells towards inflamed regions to promote repair and regeneration [87]. Furthermore it has the ability to stimulate angiogenesis. Interestingly enough, many of these restorative effects are mediated through the same receptors that mediate the pro-inflammatory properties of the molecule [88].

Obviously, the mechanisms that promote tissue structure and function restoration also include the mechanisms involved in the resolution of inflammation [89]. In particular endogenous pro-resolving lipid mediators, i.e. lipoxins, resolvins and protectins, have been the most studied [88,89]. In essence, pro-resolution factors revert back to the pro-inflammatory phenotype to its prior physiological state and therefore the microcirculatory functions of tissues and organs return to homeostasis [90].

**Figure 5:**
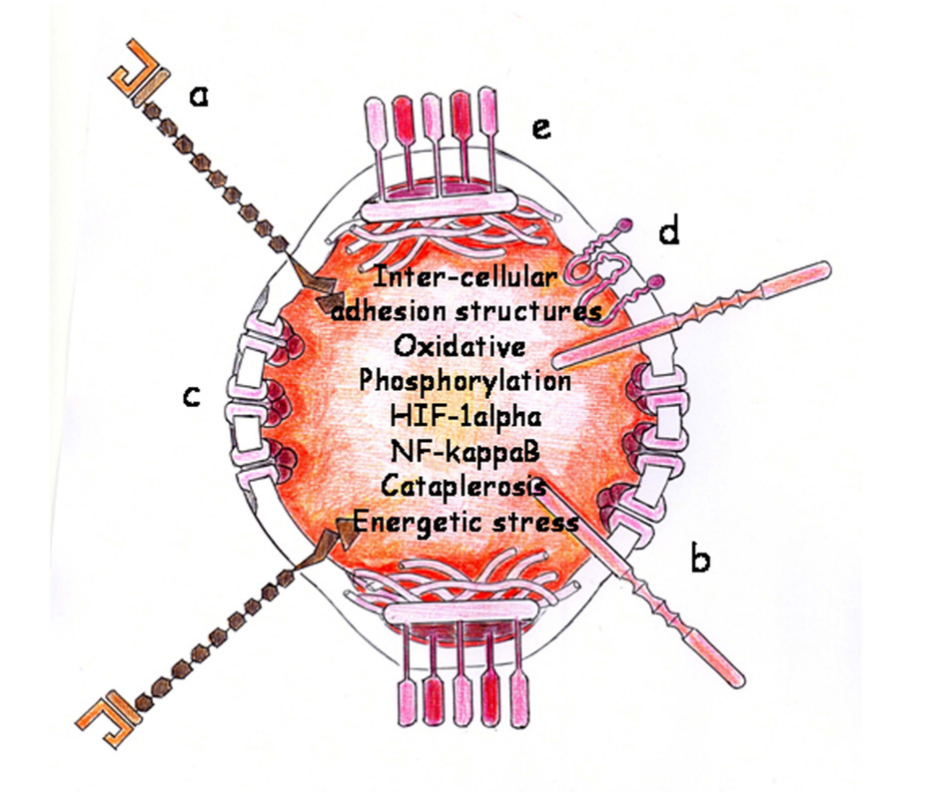
**Cellular Angiogenic phenotype**. Angiogenesis favors tissue repair as well as regeneration. a: P-Selectin; b: Integrin; c: Gap-junction; d: Claudin (tight junction protein); e: desmosome.

Nutrition mediated by blood capillaries is established because of angiogenesis. The new functional properties of microcirculation include the exchange of oxygen, nutrients and waste products. This oxygen support induces oxidative metabolism, an efficient method for extracting energy from food molecules, which begins with the Krebs cycle and ends with oxidative phosphorylation [12,13]. Oxygen and oxidative metabolism are an excellent combination through which cells can obtain an abundant energy supply (energetic stress) for tissue repair by specialized cells [12]. Nonetheless, little is known about the capacity of eukaryotic cells to monitor the redox state for supporting specialized functions [91]. Although NF-κB acts mainly as an initiator of inflammation, recent studies suggest that it also functions in the equally complex process of resolution of inflammation [92] (Figure 5).

In this convalescence phase, the hypercatabolic syndrome is progressively downregulated with the reduction of catabolic hormones and/or molecules (eg. catecholamines, pro-inflammatory cytokines, cortisol, glucagon) and the increase of anabolic hormones (eg. insulin, growth hormones, insulin-like growth factor-1 or anabolic steroids) [74,93] that are supported by tissues and organs through the new vessel arrangement and morphology. Consequently, this anabolic response counteracts catabolic stimuli and reverses muscular, both skeletal and cardiac, wasting and impaired energetic metabolism with its consequent functional damage [93]. Clinical studies in recent years have supported the concept of "immunonutrition" for severely injured patients, which takes into account the supplementation of omega-3 fatty acids and essential amino acids, such as glutamine [94].

The progressive recovery of the hypothalamic centralization of the autonomous neurofunctions (sympathetic and vagal nervous system) and endocrine (hypothalamic-pituitary-organ-hormonal axes) possibly correspond to the progressive remodeling of the tissues and organs controlled by hemodynamic and metabolic stimuli. On the contrary, leukocytes during the transition to resolution would progressively inhibit the neuroendocrine functional capacity found in the previous phases [68-70]. In turn, they would modulate this last phase of the inflammatory response when leukocytes express a lymphocytic phenotype [95]. In particular, regulatory T (Treg) cells safeguard the tissues restored against autoimmunity and immune pathology [96].

The behavior of the gastrointestinal tract under normal conditions mainly depends on the morphology and function of its microcirculation. Thus, the trophic function of the intestine is coupled with the metabolic needs of the body. In particular lymph vessels in the mucosal and submucosal layers, or initial lymphatics, recover their ability to absorb dietary fat and fat soluble vitamins, which are secreted by entorocytes in the form of lipid particles or chylomicrons [97,98].

In conclusion, the hypothetical capacity of the body to involute or dedifferentiate after a severe injury could mean an effective defense mechanism because it would make it possible to retrace a well-known metabolic route to the specialization of the systemic inflammatory response during the endocrine phase. This specialization would require the return of the prominence of oxidative metabolism and angiogenesis in the affected tissues and epithelial organs to create the capillary bed that would make the repair possible [12-14].

### The need of a metabolic staging after severe trauma

Elaborating a detailed metabolic staging after severe injury should be a preferential objective today in order to obtain a correct treatment for these patients. One requirement would be for the staging system to have a clinical significance and as a result, for it to allow for establishing the correct metabolic support.

In the current review, it has been considered that while the systemic inflammatory response develops after a severe injury, the body would successively express phenotypes of increasing metabolic complexity.

The increasing metabolic complexity of the systemic acute post-traumatic inflammatory response also shares some similarities with the successive metabolic stages that eukaryotic cells develop to obtain energy from food [99]. Therefore, in a first stage (nervous phase) macromolecules (polysaccharides, proteins and fats) are broken down by oxidative stress to smaller molecules (glucose, amino acids, triglycerides and free fatty acids). Cells with an ischemia-reperfusion phenotype would base its metabolism on anaerobic glycolysis [100]. The most important part of the second catabolic stage would be headed by enzymatic stress that produces the symbiosis of the inflammatory cells and bacteria (immune phase) [101]. Also the cells with leukocytic phenotype could use anaplerotic precursors (glutamine) to obtain energy (NADPH production) that would be employed in biosynthesis pathways (cataplerosis). The addition of an oxygen-requiring stage to the catabolic process provides cells with successively more powerful and efficient methods for extracting energy (electron transport chain). The ability to use oxygen in oxidative metabolism (oxidative phosphorylation) is recovered when the patients recover their capillary function (angiogenic phenotype) and therefore the nutrition mediated by them (endocrine or late phase). This type of metabolism is characterized by a large production of ATP (energetic stress) which is used to drive specialized multiple cellular processes with limited generation of heat.

## Conclusion

The sequence in the expression of metabolic systems that becomes progressively more elaborate and complex could be considered the essence of the metabolic evolution of severe injured patients. In the successive metabolic switches or metamorphoses that patients undergo, possibly they would retrace a well-known embryonic fetal route. If this is not properly executed, homeostasis is not recovered which is why the patient can suffer a post-traumatic stress syndrome.

The hypothesis that atmospheric oxygen concentration affected the timing of the evolution of cellular compartmentalization by constraining the size of domains necessary for communications across membranes has been suggested [102]. Thus, the relatively rapid changes in the size of the oxygen-rich external domains coincide with increasing organism complexity. This points towards a key role in the increase in abundance and size of receptors over time [102] and adds to a growing body of literature that most recently connects atmospheric oxygen levels and macroevolutionary changes with the complexity of metabolic networks and cell types [102,103]. Therefore, a correlation between increased organism complexity and the development of the use of the atmospheric oxygen could be established [104,105].

In summary, the current review about the metabolic changes developed in severe injured patients could suggest that a correlation between the different clinical phases and the corresponding metabolic stages must be established. In this way, during the Ischemia-Reperfusion phenotype expression the main objective would be to reduce the hydroelectrolytic impairments that when associated with hypometabolism favor cellular dedifferentiation. Therefore, controlled hypothermia and anaerobic glycolysis would reduce the metabolic needs of the patients and so it would be possible to diminish the deleterious effects related to ischemia-reperfusion. In this sense, fermentation would be a good metabolic pathway alternative to obtain enough energy while avoiding excessive hydroelectrolytic exchange across the cellular membranes, particularly in those tissues and organs which are more prone to this kind of injury, like intestine, kidneys and lungs [100]. During the leukocytic phenotype, the priority would be to reduce the expression of adhesion molecules and their receptors since they would induce tissue dedifferentiation. In this phase, hypermetabolism and enzymatic stress stand out. Therefore, it would be advisable to modulate the anaplerotic leukocytic metabolism to avoid uncontrolled cellular and bacterial proliferation. In addition, the early administration of enteral nutrition and the activation of the antienzymatic acute phase response would be very useful anti-inflammatory therapeutic options for severely injured patients in this evolutive phase. Finally, in the late phase associated with the angiogenic phenotype expression, probably the best measure would be the activation of the oxidative metabolism to prioritize cellular specialization with respect to proliferation. In this way, modulating angiogenesis can improve the epithelial regeneration of the injured organs, i.e., gastrointestinal tract, lungs, kidneys and liver, while avoiding the fibrotic sequelae.

One of the key challenges in future research in this clinical area would be to improve the knowledge about the exact pathophysiological mechanisms involved in the successive metabolic phenotypes described since ancient times as characteristic of the severely injured patients evolution. However, maybe this objective is not simple because the behavior of both normal and pathological organs and tissues are heterogeneous. Therefore, it would be necessary to study the metabolic relationships which are established between the different organs of the body when they suffer a severe injury. Then, the use of more organ-specific metabolic therapeutic measures would be more appropriate in the future.

## List of abbreviations

Ach: acetylcholine; ACTH: corticotropin; CARS: counter-regulatory anti-inflammatory response; CLIP: corticotropin-like intermediate lobe peptide; CO: carbon monoxide; CRF: Corticotropin releasing factor; HIF: hypoxia inducible factor; HMGB-1: high mobility group box-1; HO: heme oxygenase; H_2_S: hydrogen sulfide; LPH: β-lipotrophic hormone; MSH: γ-melanocyte-stimulating hormone; NADPH: reduced nicotinamide dinucleotide phosphate; NF-κB: nuclear factor Kappa B; NO: nitric oxide; POMC: pro-opiomelanocortin; SDF-1: stromal-derived factor; SIRS: systemic inflammatory response syndrome; Treg: regulatory T cells; VEGF: vascular endothelial growth factor.

## Competing interests

The authors declare that they have no competing interests.

## Authors' contributions

All the authors participated in the interpretation of the metabolic response to the injury based on the successive expression of three inflammatory phenotypes and helped to draft the manuscript. All authors read and approved the final manuscript.
